# Factors Associated With Progression From Level 1 to Level 2 Sensor‐Detected Hypoglycaemia in People With Type 1 and Insulin‐Treated Type 2 Diabetes: A Post Hoc Analysis From the Hypo‐METRICS Study

**DOI:** 10.1111/dom.71172

**Published:** 2026-08-04

**Authors:** Vaios Koutroukas, Aikaterina Tziannou, Jonah J. C. Thomas, Gilberte Martine‐Edith, Simon Heller, Julia K. Mader, Julie Maria Bøggild Brøsen, Ulrik Pedersen‐Bjergaard, Bastiaan E. de Galan, Eric Renard, Mark L. Evans, Rory J. McCrimmon, Jane Speight, Frans Pouwer, Stephanie A. Amiel, Pratik Choudhary

**Affiliations:** ^1^ Diabetes Research Centre University of Leicester, College of Life Sciences Leicester UK; ^2^ Cancer Research UK Clinical Trials Unit University of Birmingham England UK; ^3^ Division of Clinical Medicine, School of Medicine and Population Health University of Sheffield Sheffield UK; ^4^ Division of Endocrinology and Diabetology, Department of Internal Medicine Medical University of Graz Graz Austria; ^5^ Department of Clinical Medicine, Faculty of Health and Medical Sciences University of Copenhagen Copenhagen Denmark; ^6^ Department of Endocrinology and Nephrology North Zealand Hospital Hillerød Denmark; ^7^ Department of Internal Medicine Radboud University Medical Center Nijmegen the Netherlands; ^8^ Department of Internal Medicine Maastricht University Medical Center+ Maastricht the Netherlands; ^9^ CARIM Cardiovascular Research Institute Maastricht Maastricht University Maastricht the Netherlands; ^10^ Department of Endocrinology, Diabetes, Nutrition Montpellier University Hospital Montpellier France; ^11^ Institute of Functional Genomics, University of Montpellier Montpellier France; ^12^ Wolfson Diabetes and Endocrine Clinic Cambridge University Hospitals NHS Foundation Trust Cambridge UK; ^13^ Institute of Metabolic Science University of Cambridge Cambridge UK; ^14^ Division of Molecular and Clinical Medicine University of Dundee Dundee UK; ^15^ School of Psychology Institute for Health Transformation, Deakin University Geelong Victoria Australia; ^16^ The Australian Centre for Behavioural Research in Diabetes Diabetes Victoria Carlton Victoria Australia; ^17^ Department of Psychology University of Southern Denmark Odense Denmark; ^18^ Steno Diabetes Center Odense Odense Denmark; ^19^ Department of Health and Caring Sciences Western Norway University of Applied Sciences Bergen Norway; ^20^ Department of Diabetes, School of Cardiovascular and Metabolic Medicine and Sciences King's College London London UK

**Keywords:** continuous glucose monitoring (CGM), Hypoglycaemia, type 1 diabetes, type 2 diabetes

## Abstract

**Aims:**

We investigated the proportion of sensor‐detected hypoglycaemic (SDH) events progressing to level 2, and associated variables.

**Materials and Methods:**

We used data from Hypo‐METRICS, which recruited people with type 1 (pwT1D) and insulin‐treated type 2 diabetes (pwT2D) with ≥ 1 hypoglycaemic event in preceding 3 months, wearing blinded continuous glucose monitoring devices (CGM), additional to usual monitoring modality, and FitBits for 10 weeks. We defined:

L1: SDH < 3.9 mmol/L for ≥ 15 min but ≥ 3.0 mmol/L;
L2: SDH < 3.9 mmol/L with ≥ 15 min of sensor glucose < 3.0 mmol/L;
L2 ratio = L2/(L1 + L2), restricted to participants with both event types during the study period.

Associations of selected variables on L2 ratio was assessed using beta regression and purposeful variable selection.

**Results:**

We analysed 23 768 SDH events from 212 pwT1D and 213 pwT2D. PwT1D were predominantly female (53% vs. 42% in T2D, *p* = 0.023) and younger (median age 49 vs. 62 years, *p* < 0.001), with a higher L2 ratio (16% vs. 14%, *p* = 0.03). Coefficient of variation (CV), OR = 1.07, 95% CI = 1.06–1.09 (*p* < 0.001), and mean sensor glucose, OR = 1.13, 95% CI = 1.08–1.18 (*p* < 0.001) were the principal predictors in T1D and T2D respectively; mean L1‐SDH duration, weekly L1‐SDH frequency, personal CGM usage, impaired awareness were not. Progression was higher during sleep than wakefulness (21% vs. 11%, *p* < 0.001); L2 ratios during sleep did not differ by awareness status.

**Conclusions:**

Approximately one sixth of SDH events progressed to L2, with a higher proportion in T1D than T2D. The principal determinants were CV in T1D and mean glucose in T2D. Awareness status was not associated with progression risk during sleep.

## 1 Introduction

Hypoglycaemia is a limiting factor in optimising glucose levels in insulin‐treated diabetes [1] to prevent long‐term complications [2]. The International Hypoglycaemia Study Group (IHSG) defines hypoglycaemia as level 1 (L1), glucose ≥ 3.0 mmol/L and < 3.9 mmol/L; level 2 (L2), glucose < 3.0 mmol/L; level 3 (L3) or severe hypoglycaemia (SH), associated with cognitive impairment requiring external assistance for recovery [3].

L1, an alert for hypoglycaemia, denotes the need for treatment to prevent further glucose decrease, with symptomatic events having a negative impact on quality of living [4]. L1 [5, 6] but, mainly, L2 hypoglycaemia [5, 6], is associated with impaired cognitive function, inflammation, increased cardiovascular risk, and impaired awareness of hypoglycaemia (IAH). L2 events are also associated with increased mortality in people with type 2 diabetes (pwT2D) [7, 8] and those in critical care environments [9], and are therefore termed clinically important [3]. Most events remain at L1, recovering either spontaneously or because of treatment. However, a proportion of L1 events progresses to the clinically important L2.

Diminution of counterregulatory (CRR) and symptom responses to hypoglycaemia, increasing insulin deficiency [10], age, and diabetes duration may amplify progression risk from L1 to L2 [11, 12]. Counterregulatory and symptom responses are reduced during sleep [13], and in IAH, an independent risk factor for SH [14] even in the continuous glucose monitoring (CGM) era [15, 16].

The extent to which L1 hypoglycaemia progresses to L2 in clinical practice has not been quantified. We explored factors that are associated with the proportion of sensor‐detected hypoglycaemic (SDH) events progressing from L1 to L2 in pwT1D and pwT2D. We then investigated the impact of sleep/awake status on that progression, exploring differences in the overall sample and by subgroups.

## 2 Materials and Methods

### 2.1 Study Design

Hypoglycaemia—MEasurement, ThResholds and ImpaCtS (Hypo‐METRICS) was a 10‐week observational study conducted across nine U.K. and EU sites between October 2020 and August 2022, as part of the Hypo‐RESOLVE project [17].

### 2.2 Study Trial Population

Hypo‐METRICS recruited 602 adults (18–85 years) with T1D and insulin‐treated T2D taking ≥ 1 insulin injection daily, having experienced ≥ 1 hypoglycaemic episode in the 3 months prior to study enrolment. Diabetes type was determined based on clinical features. Key exclusion criteria were an estimated glomerular filtration rate of < 30 mL/min/1.73 m^2^ and automated insulin delivery (AID) use. Participants wore a blinded CGM device (Abbott Freestyle Libre 2) and a sleep and activity tracking device (Fitbit Charge 4) continuously for 10 weeks. CGM data were collected at 5‐min intervals and were interpolated for 1‐min data [18]. Participants continued glucose monitoring, CGM or self‐monitoring blood glucose (SMBG), as before enrolment; those on CGM as their usual monitoring modality wore both their personal and the study blinded sensors during the study period.

*Hypoglycaemia awareness* status was assessed at enrolment with the Gold question [19]; normal awareness (NAH) was defined as Gold ≤ 3.

### 2.3 Asleep/Awake Status

The Fitbit Charge 4 detected sleep periods combining accelerometry and heart rate data, without requiring manual input from participants [20]. A SDH event was classified as “whilst asleep” if it occurred between start and end time of sleep (including daytime sleep) as identified by Fitbit, and “whilst awake” if it occurred outside sleep intervals. If a SDH event overlapped between asleep/awake periods, it was attributed the sleep status for which the area under the curve was the greatest during sleep [21].

### 2.4 Definition of SDH Levels

**FIGURE 1:**
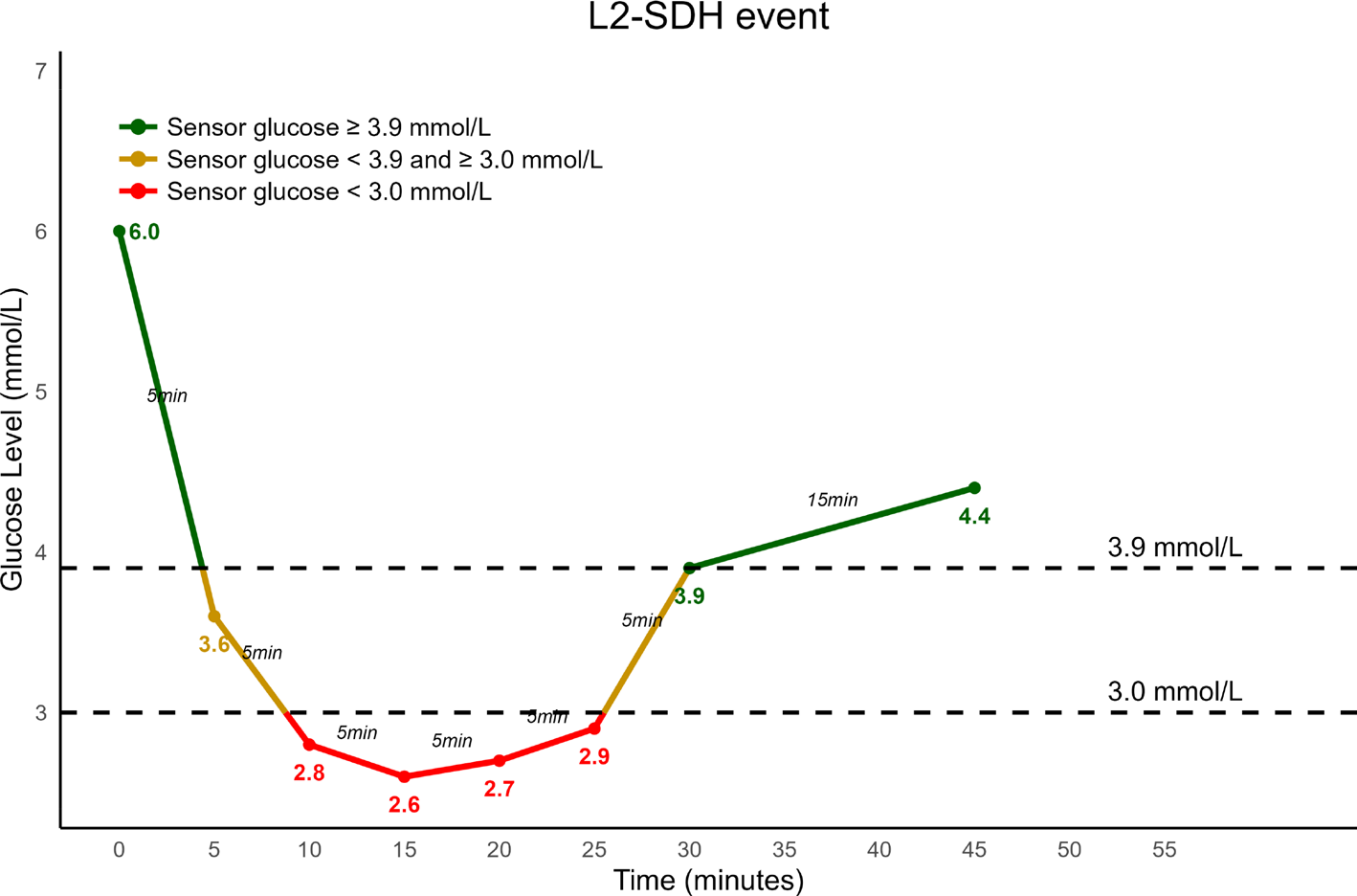
Schematic representation of a Level 2 sensor‐detected hypoglycaemic (L2‐SDH) event. Glucose falls below 3.9 mmol/L at 5 min and below 3.0 mmol/L at 10 min, remaining < 3.0 mmol/L for 15 min (10–25 min), before recovering to ≥ 3.9 mmol/L and remaining there for ≥ 15 min (30–45 min), at which point the event is considered resolved. The event is classified as L2‐SDH because it contains ≥ 15 min of glucose < 3.0 mmol/L.

*SDH < 3.9 mmol/L Events*: These include all events with sensor glucose < 3.9 mmol/L for ≥ 15 min, divided into:

*L2* events, which contained ≥ 15 min of glucose < 3.0 mmol/L (Figure 1).

**FIGURE 2:**
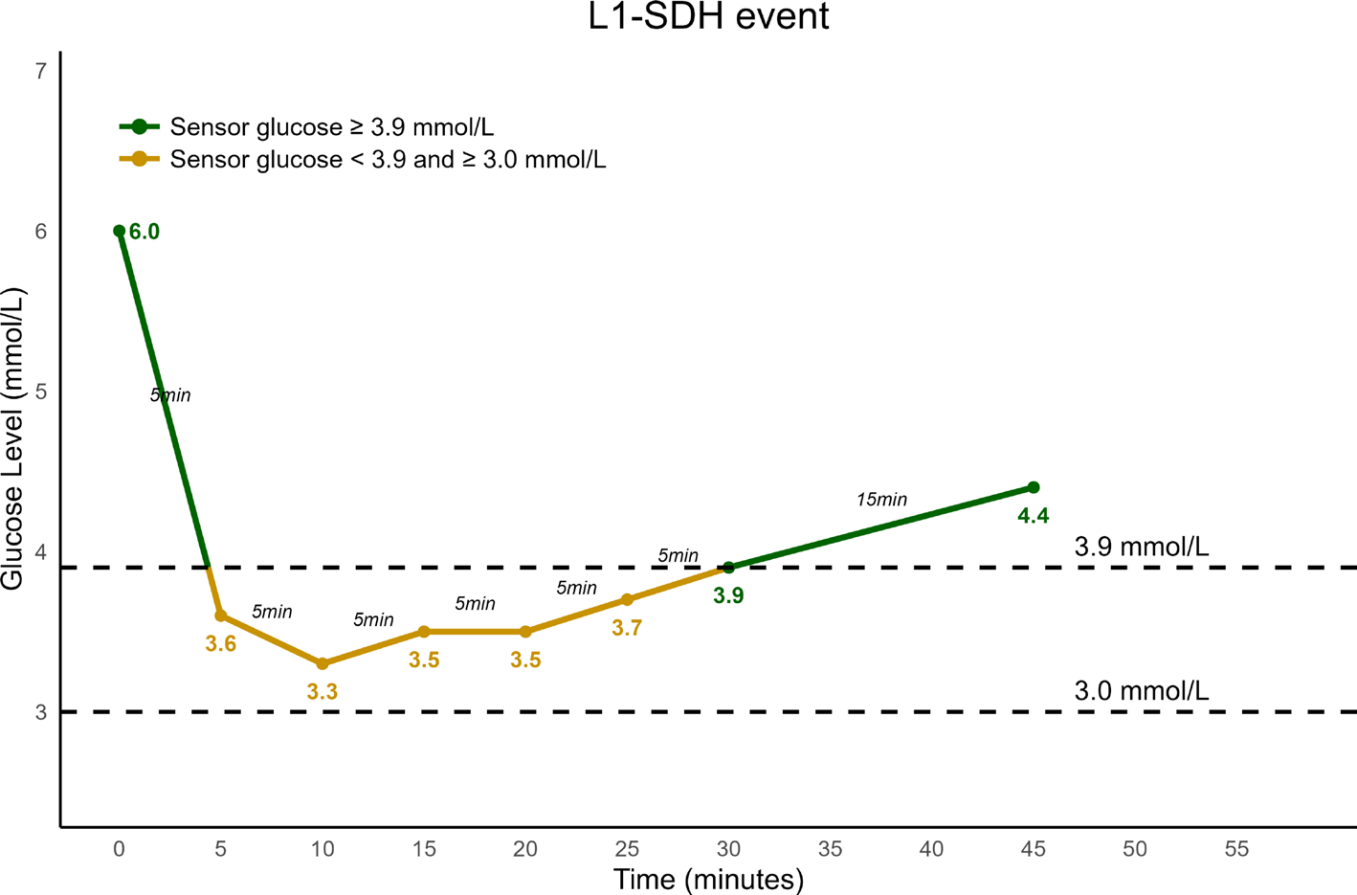
Schematic representation of a Level 1 sensor‐detected hypoglycaemic (L1‐SDH) event. Glucose falls below 3.9 mmol/L at 5 min but remains ≥ 3.0 mmol/L throughout, recovering to ≥ 3.9 mmol/L at 30 min and remaining there for ≥ 15 min (30–45 min), at which point the event is considered resolved. The event is classified as L1‐SDH because it does not contain any period of ≥ 15 min with glucose < 3.0 mmol/L.

*L1* events, which did not contain any period of ≥ 15 min with glucose < 3.0 mmol/L. Brief excursions < 3.0 mmol/L could occur, provided they did not meet the minimum duration criterion for L2 classification (< 15 min) (Figure 2).

*Resolution* of either L1‐SDH or L2‐SDH was defined as ≥ 15 consecutive minutes with ≥ 2 sensor glucose values ≥ 3.9 mmol/L for ≥ 15 min apart, with no intervening values < 3.9 mmol/L.

*L2 ratio* = (L2‐SDH)/(total‐SDH); total‐SDH: aggregate of L1‐SDH and L2‐SDH events.

### 2.5 Study Sample

Only participants with > 70% active data from > 14 days of blinded CGM wear were included in the analysis [22].

### 2.6 Collected Variables

The following variables were considered clinically relevant to L1 → L2 SDH progression:

*Person‐derived*: age (years; median), sex (male/female; count and percentage), race (white/non‐white; count and percentage), and hypoglycaemia awareness status (count and percentage).

*CGM‐derived*: mean sensor glucose (mmol/L; reported as median); coefficient of variation (CV, %; median), glucose monitoring modality (CGM or SMBG; count and percentage), referring to participants' usual monitoring practice prior to study enrolment; mean L1‐SDH duration/participant (minutes; reported as median), weekly mean L1‐SDH frequency/participant (reported as median).

### 2.7 Statistical Analysis

Participants' baseline characteristics were computed via a descriptive analysis. Data normality was assessed by histograms and the Kolmogorov‐Smirnoff test. The cohort was subdivided by diabetes type (T1D, T2D) and analyses were performed in each group (T1D + T2D, T1D, T2D) separately.

Beta regression models with a logit link were used to assess the associations of the collected variables with L2 ratio across each participant's observation period. The distribution of SDH events/participant was examined to assess L2 ratio reliability. Only participants with ≥ 1 L1‐SDH and ≥ 1 L2‐SDH event were analysed. We chose beta over linear regression because of our outcome variable (ratio), and its highly skewed distribution [23]. Participants with no L2‐SDH events were excluded as beta regression requires the outcome to lie strictly within (0,1): the analysis of those participants' L1‐SDH events would constitute a separate research question beyond the scope of this study. Predictors of the L2 ratio were identified using purposeful variable selection [24], comprising univariable screening, multivariable model building, and confounding assessment. Multicollinearity was tested using variance inflation factor, with < 5 indicating no substantial multicollinearity [25]. Results were presented as exponentiated coefficients, interpreted analogously to odds ratios (95% CIs, *p*‐values).

We then compared L1 → L2 progression ratios during sleeping versus waking hours with non‐parametric tests, to account for the skewness of our data. Only participants with SDH events whilst both asleep/awake were included. Wilcoxon signed‐rank tests were used for within‐participant comparisons (asleep vs. awake). Between‐group comparisons (T1D vs. T2D; NAH vs. IAH; CGM vs. SMBG users) were conducted using Wilcoxon rank‐sum tests. We applied the Benjamini‐Hochberg (BH) correction for multiple comparisons. Between‐group analyses were exploratory in nature and are interpreted accordingly.

A 5% significance level was used throughout; all analyses were performed in R version 4.4.2 [26].

## 3 Results

### 3.1 Study Population and Distribution of SDH Events

From 602 participants recruited, 425 (212 pwT1D, 213 pwT2D) were included (55 did not consent for their data to be used in secondary analyses; 49 had not completed the Gold question or did not have > 70% active data from > 14 days of blinded CGM wear; 70 had no L2‐SDH events; 2 had no reported L1‐SDH events; 1 had recorded their sex as other than male or female). PwT1D were predominantly female (53% vs 42% in T2D, *p* = 0.023) and younger (median 49 vs. 62 years, *p* < 0.001). Although AID users were excluded, open loop pump users were included (Table S1).

**FIGURE 3:**
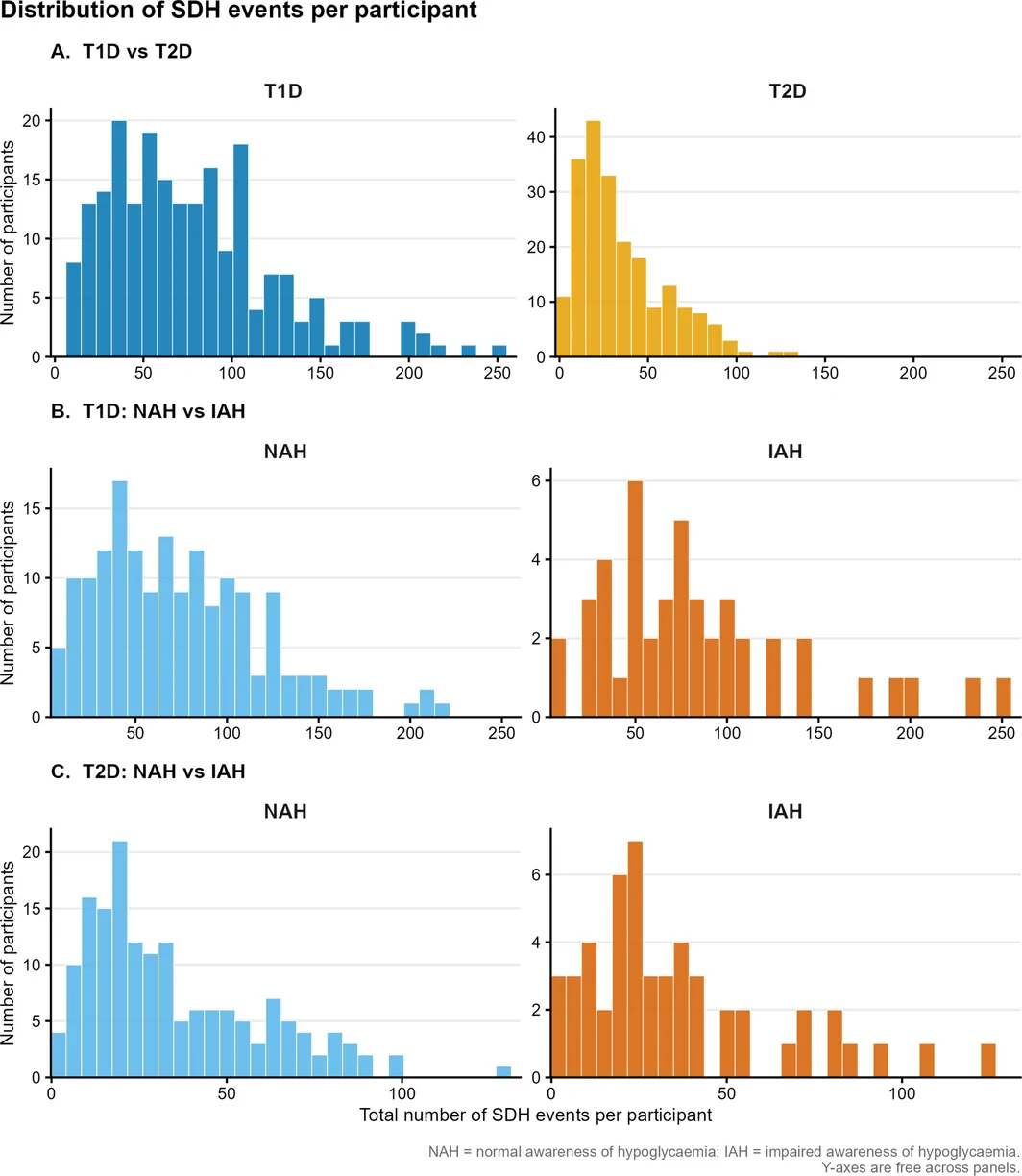
Distribution of total sensor‐detected hypoglycaemia (SDH) events per participant. Panel A: T1D versus T2D. Panels B and C: T1D and T2D respectively, stratified by hypoglycaemia awareness status. *Y*‐axes are free across panels. Note the difference in scale between T1D and T2D, and between NAH and IAH subgroups. NAH, normal awareness; IAH, impaired awareness.

We analysed 23 768 SDH events (16 304 in T1D; 7464 in T2D). The median number of total episodes/participant was 47 (IQR = 24–85; range = 2–251). PwT1D had a higher number of events compared with pwT2D: 69 (IQR = 41–103; range = 8–251) versus 27 (IQR = 17–47; range = 2–129), *p* < 0.001. In pwT1D, median total episodes/participant with NAH were 67 (IQR = 39–103; range = 8–216) vs 75 (IQR = 46–103; range = 8–251) in those with IAH, *p* = 0.41; among pwT2D, median total episodes/participant with NAH were 27 (IQR = 16–49; range = 2–129) and 27 (IQR = 18–46; range = 2–126) in those with IAH, *p* > 0.9 (Figure 3).

PwT1D had a similar, albeit shorter, mean L1‐SDH duration (49 min vs 52 min, *p* = 0.002), a higher weekly mean L1‐SDH frequency (5.7 vs. 2.4 events/week, *p* < 0.001), and a higher L2 ratio (16% vs. 14%, *p* = 0.03) to pwT2D; mean sensor glucose (8.6 vs. 8.5 mmol/L, *p* = 0.50) was similar (Table S1). In T1D, individuals with IAH were older and had longer diabetes duration; in T2D, those with IAH were younger and had shorter diabetes duration than those with NAH (Table S1).

### 3.2 Purposeful Variable Selection Results

**FIGURE 4:**
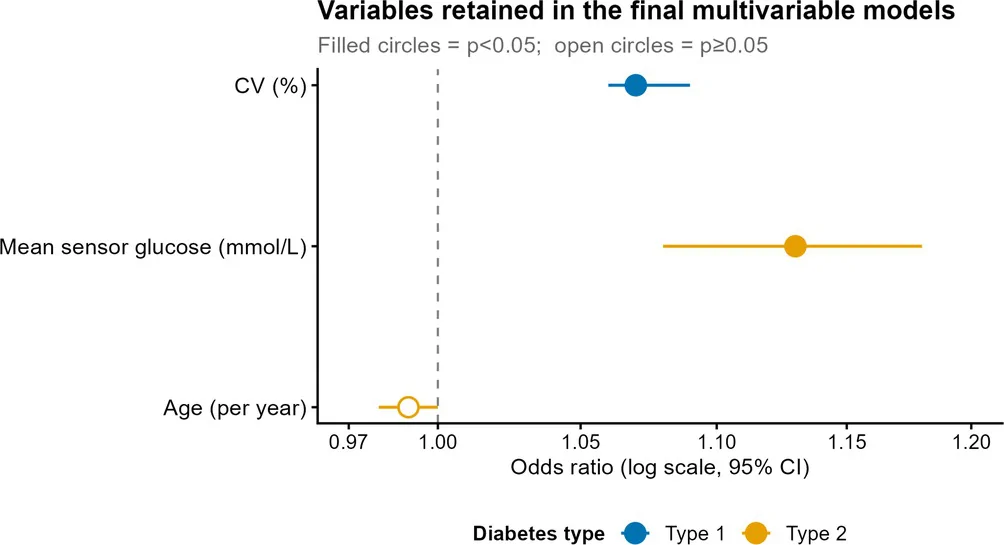
Variables retained in the final multivariable beta regression models for type 1 and type 2 diabetes. Odds ratios (ORs) with 95% confidence intervals are presented on a log scale. In type 1 diabetes, coefficient of variation (CV) was the sole independent predictor of the L2 ratio. In type 2 diabetes, mean sensor glucose was the primary independent predictor, with age retained as a clinically relevant confounder. Filled circles indicate *p* < 0.05; open circles indicate *p* ≥ 0.05. The dashed vertical line represents OR = 1. Blue = type 1 diabetes; amber = type 2 diabetes.

In the combined model, higher CV was independently associated with greater L1 → L2 progression (OR = 1.04 per 1% increase, 95% CI = 1.03–1.05, *p* < 0.001), as was IAH (OR = 1.20, 95% CI = 1.04–1.39, *p* = 0.014). Mean L1‐SDH duration, weekly mean L1‐SDH frequency, diabetes type, age, mean glucose, and glucose monitoring modality were not retained as independent predictors. Sensitivity analysis excluding influential observations confirmed model stability, with all key coefficients changing by < 10% (Figure 4).

**FIGURE 5:**
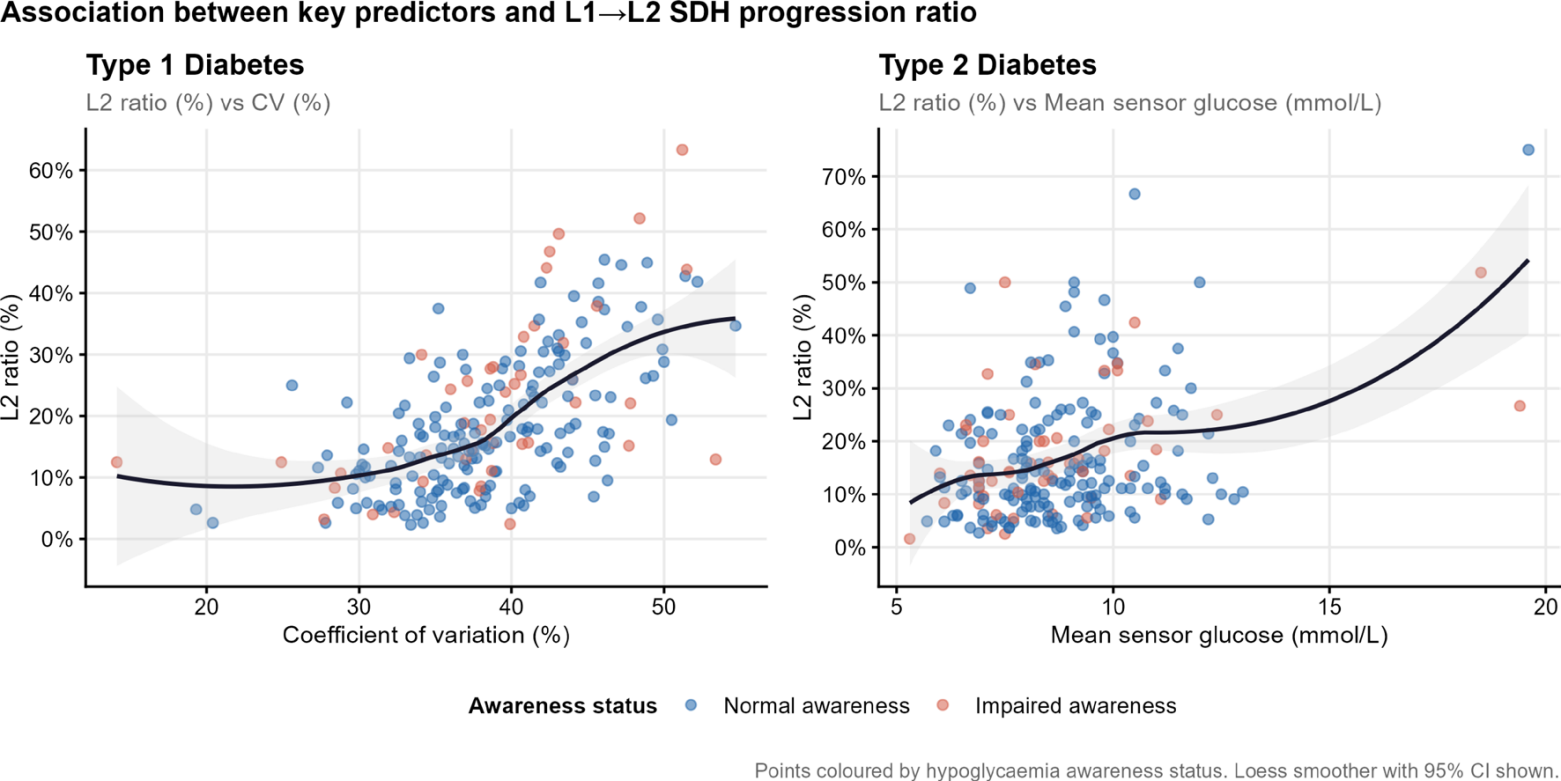
Association between key glycaemic predictors and the L1 → L2 SDH progression ratio, by diabetes type. Left panel: Coefficient of variation (CV%) versus L2 ratio in T1D (*n* = 212). Right panel: Mean sensor glucose vs. L2 ratio in T2D (*n* = 213). Points are coloured by hypoglycaemia awareness status as assessed by Gold measure. The curve represents a locally estimated scatterplot smoothing (LOESS) line with 95% confidence interval.

In T1D, CV (Figure 5) was the sole independent predictor of progression (OR = 1.07 per 1% increase, 95% CI = 1.06–1.09, *p* < 0.001). Sensitivity analysis confirmed its stability (13.7% change).

In T2D, higher mean sensor glucose (Figure 5) was the principal independent predictor of progression (OR = 1.13 per 1 mmol/L increase, 95% CI = 1.08–1.18, *p* < 0.001), with a borderline inverse association for age (OR = 0.99 per year, 95% CI = 0.98–1.00, *p* = 0.053). Sensitivity analysis showed that the mean glucose coefficient changed by 23.5%, reflecting the influence of a small number of participants with high L2 ratios and elevated mean glucose.

### 3.3 SDH Progression Rate Results During Sleeping Versus Waking Hours, Subgrouped by Diabetes Type, Glucose Monitoring Modality, and Awareness Status

Data from 399 participants (204 pwT1D, 195 pwT2D) were analysed; 26 were additionally excluded due to missing asleep/awake classification or absence of SDH events in both states, to preserve valid intraindividual comparisons.

Among 21 147 SDH events (14 644 in pwT1D; 6503 in pwT2D), 26% occurred during sleep (T1D: 22%; T2D: 37%). L2 ratios were higher during sleep than wakefulness irrespective of diabetes type or glucose monitoring modality (Table S2): median 21% (Q1–Q3 = 10%–33%) vs. 11% (Q1–Q3 = 3%–20%), BH‐adjusted *p* < 0.001. This pattern was consistent in both NAH [21% (Q1–Q3 = 10%–32%) vs. 10% (Q1–Q3 = 3%–20%), BH‐adjusted *p* < 0.001] and IAH subgroups [24% (Q1–Q3 = 11%–40%) vs. 13% (Q1‐Q3 = 4%–22%), BH‐adjusted *p* < 0.001], with two exceptions: no significant sleep/wake difference was observed in SMBG users with T1D (BH‐adjusted *p* > 0.9) or pwT2D using CGM (BH‐adjusted *p* = 0.45).

Comparisons between NAH and IAH showed no statistically significant differences in L2 ratios during sleep or wakefulness after BH adjustment, irrespective of diabetes type or glucose monitoring modality (all BH‐adjusted *p* ≥ 0.2; Table S3). Although L2 ratios were numerically higher in IAH than NAH across all SDH events (17% vs. 14%, *p* = 0.038), this did not survive BH correction (*p* = 0.2). Within T1D, no between‐awareness comparisons reached significance after BH correction (all *p* = 0.18–0.48). In T2D, no comparisons approached significance before or after adjustment (all BH‐adjusted *p* ≥ 0.34).

## 4 Discussion

In this post hoc study, we found that among participants who experienced both L1‐SDH and L2‐SDH events, one sixth became L2, with a higher proportion seen in pwT1D than pwT2D. In the combined model (T1D + T2D), CV and IAH were independently associated with greater progression. Glycaemic variability, as quantified by CV, was the sole independent predictor of progression in T1D, while higher mean sensor glucose was the principal predictor in T2D. Mean L1‐SDH frequency, mean L1‐SDH duration, personal CGM usage, and diabetes type were not retained as independent predictors in any model. Progression was more common during sleep irrespective of diabetes type, although no difference was found in SMBG users with T1D and IAH, or in CGM users with T2D and IAH; in neither was it affected by hypoglycaemia awareness status.

### 4.1 Determinants of Hypoglycaemia Progression

In T1D, CV was identified as the sole predictor of L1 → L2 SDH progression. Greater glycaemic variability has been associated with higher risk of hypoglycaemia [27, 28, 29], and higher CV may reflect greater glucose fluctuations increasing the likelihood of a L1‐SDH event becoming L2‐SDH, particularly in T1D where CRR is more frequently impaired. In 2024, with data from a real‐world study, Brett McQueen and associates described a lower proportion of L2 events with decreasing CV levels in PwT1D [30]. We additionally explored standard deviation (SD)—the other most commonly reported CGM‐derived variability metric in clinical practice—as an alternative measure in a sensitivity analysis, as SD has been proposed as a potentially more direct SDH progression predictor [31]. This did not alter the main findings in either T1D or T2D. However, in the combined model, SD alongside mean glucose introduced collinearity and instability of the mean glucose coefficient, supporting the robustness of CV as the primary variability metric.

Neither mean L1‐SDH duration nor weekly mean L1‐SDH frequency were retained as independent predictors, T1D or T2D. Antecedent hypoglycaemia at < 2.8 mmol/L induces defective glucose counterregulation and reduced symptom perception [32], and greater L2‐SDH exposure has been associated with a blunted adrenaline response in clamp studies in pwT1D [33]. Impaired CRR to clamp‐induced hypoglycaemia has also been demonstrated following antecedent L1 events, not exclusively L2 [34]. Nevertheless, this effect has been described most consistently at L2, and the clinical definitions reflect this: the IHSG defines L1‐SDH as an alert value [3] not as a clinically important outcome in itself. Similarly, the FDA's draft guidance on efficacy endpoints for antihypoglycaemic agents proposes L2 and L3—but not L1—as acceptable endpoints for hypoglycaemia treatment [35]. It should also be noted that the existing evidence derives predominantly from mechanistic studies, whilst our findings are based on everyday life CGM hypoglycaemic exposure.

Higher mean sensor glucose in T2D was associated with greater progression risk. This could be explained by insulin stacking, namely successive corrective doses potentially amplifying glucose nadirs [36]. This can result in a steeper rate of change, leading to a greater proportion of L2 events. However, the reverse association is equally plausible: L2 events themselves may lead to subsequent hyperglycaemia through carbohydrate intake and CRR: a recent cross‐sectional study demonstrated that pwT1D experiencing rebound hyperglycaemia following nocturnal hypoglycaemia had both higher L2 event frequency and higher mean glucose levels than those without [37]. The observational design of the study precludes determination of the direction of this association. It is also worth noting that the mean glucose coefficient showed the largest change in sensitivity analysis (23.5%), driven by a small number of pwT2D with disproportionately high L2 ratios and elevated mean glucose; caution is warranted in interpreting the magnitude of this association.

In T2D, an unexpected finding was that individuals with IAH were younger and had shorter diabetes duration compared to those with normal awareness. This contrasts with the pattern observed in T1D and with established physiological expectations, reflecting differences in clinical or behavioural characteristics in pwT2D, such as earlier adoption of intensive insulin therapy or differing thresholds for symptom recognition. The difference could also be due to survival bias: older pwT2D may have mitigated progression risk through more conservative glucose targets and consistent routines.

### 4.2 Sleep as a Determinant of L1 → L2 Progression

Across both diabetes types, 21% of all SDH events progressed to L2 during sleep versus 11% while awake. Intuitively, people may be more able to perceive and respond to falling glucose levels when awake; this finding is consistent with earlier physiological studies showing that CRR is attenuated during sleep in pwT1D, including blunted secretion of adrenaline and cortisol [38], albeit less so in pwT2D [39].

Even among participants with NAH, progression rates during sleeping hours were substantially higher (21% vs. 10%), in keeping with evidence that both autonomic and neuroglycopenic responses to hypoglycaemia vary by sleep stage in healthy subjects [40], being attenuated during late sleep [13]. Clinically, this underscores the need to minimise sleep disruption as a result of hypoglycaemia or otherwise. AID systems have been shown to reduce overnight hypoglycaemia without worsening overall glycaemic control [41, 42]. These systems are currently available only for pwT1D; problematic hypoglycaemia during sleep could represent an indication for AID therapy in pwT2D in the future.

### 4.3 IAH, CGM Usage

In the subgroup models, awareness status did not independently predict progression in either T1D or T2D. This contrasts with prior research associating awareness status with overall SH risk [43], suggesting that awareness status may influence total hypoglycaemia exposure rather than per‐event progression probability. Notably, IAH was retained as an independent predictor in the combined model (T1D + T2D), where greater statistical power may have enabled detection of a more modest effect.

Personal CGM usage was not identified as a predictor of L1 → L2 SDH progression either. Possible explanations could be behavioural delays in hypoglycaemia treatment, or associated psychological burden, thus allowing L1 events to progress into more serious ones. We know that in children and adolescents with T1D, even when CGM alarms are triggered, up to 29% of nocturnal events do not cause awakening of the individual, with 66% of them only awakening after successive alerts [44]. The blinded study sensors used in Hypo‐METRICS had no active alarms; however, it is possible that some participants using CGM in routine care transitioned to alarm‐enabled devices during the study period (2020–2022), and this cannot be fully excluded as a confounding influence. Nevertheless, the substantially higher progression rates during sleep observed here are consistent with physiological attenuation of CRR during sleep itself [13].

Between those with IAH versus NAH during sleep, CGM users with T1D represented the only subgroup to have had a higher L2 ratio; after applying BH correction, awareness status had no impact on L2 ratio during sleep, irrespective of diabetes type or glucose monitoring modality. This may represent a selection bias: CGM users may have already had more problematic hypoglycaemia, including those with IAH; they had thus been prioritised for CGM, subsequently lowering their hypoglycaemic burden.

Between sleeping versus waking hours, the progression rate was greater during sleep with two exceptions: SMBG users with T1D and IAH, and CGM users with T2D and IAH. It could be that, because these two subgroups had much fewer participants than the rest, there were fewer SDH events for analysis, which may not have sufficed to highlight any statistically significant differences.

### 4.4 Strengths

The Hypo‐METRICS dataset had one million hours of CGM data. The use of a validated wrist‐worn device to objectively classify sleep status enabled estimation of hypoglycaemia progression rates during sleep. The application of beta regression modelling, appropriate for bounded ratio outcomes, ensured valid identification of independent predictors.

### 4.5 Limitations

Our inclusion criterion of hypoglycaemia in the previous 3 months may have enriched the sample, particularly in the insulin‐using T2D population, where a smaller proportion of individuals typically experiences hypoglycaemia [45]. The observational design precludes causal inference, and residual confounding from insulin dosing, meal timing, and physical activity cannot be ruled out; we did not have data on insulin doses or dose timing. Factors not captured in this study—including chronotype, brief nocturnal awakenings, and time spent in each sleep stage—may have influenced the sleep‐related findings. Exclusion of AID users limits generalisability to the most technologically advanced population.

Awareness status was assessed using the Gold measure; we have recently shown that different measures of awareness identify different individuals with IAH, and the absence of a significant association between awareness and L2 ratio may partly reflect the limitations of this instrument rather than a true absence of effect [46]. Interstitial glucose measurements inherently lag plasma glucose [47], potentially underestimating the rapidity of L1 → L2 transitions. While the blinded study sensor had no active alarms, the potential influence of alarms from participants' personal glucose monitoring devices during the study period was not captured and cannot be fully excluded. People who activate CGM alarms may have different personal or glycaemic characteristics from those who do not, a potential confounding source that was not accounted for in this analysis [48]. Some SDH events may reflect sensor inaccuracy, compression artefacts, or exercise‐related glucose falls; however, these are expected to represent a small proportion of the over 21 000 events analysed and would be unlikely to systematically bias between‐group comparisons. The data were collected in a predominantly white European population using a single sensor (FreeStyle Libre 2; mean absolute relative difference 9.2% [49]), and findings may not generalise fully to more diverse populations or other sensor systems.

A small proportion of participants contributed few hypoglycaemic events: 7.1% of pwT1D and 12.2% of pwT2D had fewer than 10 episodes. Removal of these participants did not change the core findings. Factors associated with the occurrence of L2‐SDH among those who never experienced such events represent an important question for future research. Participants with higher mean glucose in T2D may have received intensive insulin regimens, which could have partially confounded the observed association with the L2 ratio; insulin data were partially captured via free text, precluding reliable stratification by treatment modality. Body mass index, previous severe hypoglycaemia, and renal function were either incompletely captured or excluded by design, and their potential confounding effects cannot be fully excluded either.

## 5 Conclusion

This analysis demonstrates that, among participants who experienced both L1‐SDH and L2‐SDH events, progression from alert‐level to clinically important hypoglycaemia affects approximately one sixth of SDH events, with a higher proportion in T1D than T2D. The principal determinants differ by diabetes type: glycaemic variability, as quantified by CV, was the sole independent predictor in T1D, while higher mean sensor glucose was the principal predictor in T2D. IAH was independently associated with greater progression in the combined model. Sleep substantially heightens progression risk across both diabetes types, independently of glucose monitoring modality or awareness status. These findings suggest that the underlying glycaemic environment—CV in T1D, mean glucose in T2D—determines the probability of progression to clinically important hypoglycaemia, rather than alert‐level events per se. Longitudinal analyses are warranted to determine whether a higher L1 → L2 SDH progression ratio predicts subsequent development of IAH or risk of severe hypoglycaemia. If validated, the L1 → L2 SDH progression ratio could serve as a novel biomarker for individual risk stratification.

## Author Contributions

V.K. and P.C. developed the plan for the manuscript, with input and advice from the remaining coauthors. V. K. generated the tables and the figures, and prepared the manuscript draft, with critical feedback from the remaining coauthors. V. K. and A.T. conducted the analysis. J.J.C.T, G.M.‐E., A.T., S.H., J.K.M., J.M.B.B., U.P.‐B., B.E.d.G., E.R., M.L.E., R.J.M., J.S., F.P., S.A.A., and P.C. prepared subsequent revisions with feedback from all coauthors. All authors approved the final draft of the manuscript. V.K. is the guarantor of this work and, as such, had full access to all the data in the study and takes responsibility for the integrity of the data and the accuracy of the data analysis.

## Funding

This work was supported by the Innovative Medicines Initiative 2 Joint Undertaking (JU) under grant agreement 777460. JU receives support from the European Union's Horizon 2020 Research and Innovation Programme and European Federation of Pharmaceutical Industries and Associations, and T1D Exchange, Juvenile Diabetes Research Foundation, International Diabetes Federation, and The Leona M. and Harry B. Helmsley Charitable Trust. The industry partners supporting JU include Abbott Diabetes Care, Eli Lilly, Medtronic, Novo Nordisk, and Sanofi‐Aventis. Abbott Diabetes Care provided the continuous glucose monitors used in the study. The views expressed are those of the author(s) and not necessarily those of the National Health Service, NIHR, the Department of Health and Social Care or the JU.

## Conflicts of Interest

V.K. has received funding from KelCon GmbH to attend ATTD 2024. He is also the recipient of YDEF‐Lilly scholarships to attend EASD 2024 and ATTD 2026, and attended the EASD Robert Turner course 2025, partially funded by Lilly. J.J.C.T. has no disclosures to declare. G.M.‐E. has no disclosures to declare. A.T. has no disclosures to declare. S.H. undertakes consultancy with Eli Lilly, NovoNordisk, Zealand Pharma, Vertex, Zucara, Medtronic and receives research support from Dexcom Inc. J.K.M. is a member of advisory boards of Abbott Diabetes Care, Becton‐Dickinson, Biomea Fusion, Dexcom, Eli Lilly, Embecta, Insulet, Medtronic, Novo Nordisk A/S, Pharmasens, Roche Diabetes Care, Sanofi‐Aventis, Tandem. She has received speaker honoraria from A. Menarini Diagnostics, Abbott Diabetes Care, Buzud, Dexcom, Embecta, Eli Lilly, Novo Nordisk A/S, Roche Diabetes Care, Sanofi, Sinocare, Viatris and Ypsomed. She is also a shareholder of decide Clinical Software GmbH and elyte Diagnostics and serves as CMO of elyte Diagnostics. J.M.B.B. received a one‐time lecture fee from Boehringer Ingelheim and travel support (flight and hotel) for EASD 2024 from AstraZeneca. U.P.‐B. has received consulting fees from Abbott, support for attending conferences from Novo Nordisk and Sanofi, and trial supplies from Novo Nordisk. B.E.d.G. has no disclosures to declare. E.R. declares consultant/speaker fees from A. Menarini Diagnostics, Abbott, Air Liquide SI, Astra‐Zeneca, Becton‐Dickinson, Boehringer‐Ingelheim, Cellnovo, Dexcom Inc., Eli‐Lilly, Hillo, Insulet Inc., Johnson & Johnson (Animas, LifeScan), Medtronic, Medirio, Novo‐Nordisk, Roche, and Sanofi‐Aventis and research support by Abbott, Dexcom Inc., Insulet Inc., Roche, and Tandem Diabetes Care. M.L.E. has received personal fees from Abbott Diabetes Care, Eli Lilly and Company, Medtronic, Dexcom, Novo Nordisk, Astra Zeneca, and Zucara. The University of Cambridge has received salary support for M.L.E. from the National Health Service in the East of England through the Clinical Academic Reserve. R.J.M. has no disclosures to declare. J.S. reports honoraria (to research group) from Sanofi and Vertex for service on advisory boards. F.P. has received funding for research from Novo Nordisk, Eli Lilly, and Sanofi‐Aventis. S.A.A. has served on an advisory board for Vertex Pharmaceuticals and has spoken at educational symposia sponsored by Vertex and by Sanofi. No other potential conflicts of interest relevant to this article were reported. P.C. has received personal fees Abbott Diabetes Care, Insulet, Dexcom, Novo Nordisk, AstraZeneca, Medtronic, Roche Diabetes Care, and Sanofi Diabetes and research funding support from Abbott Diabetes Care, Medtronic, and Novo Nordisk.

## Supporting information


**Table S1:** Characteristics of the study population.
**Table S2:** Difference in the L2 ratio between sleeping and waking hours within the total sample (T1D + T2D, T1D, and T2D), subsectioned by modality of glucose monitoring (CGM + SMBG, CGM, and SMBG users), and awareness status (NAH + IAH, NAH, IAH). Benjamini‐Hochberg (BH) correction has been applied for multiple comparisons. *p* ≥ 0.05 have been underlined.
**Table S3:** Difference in the L2 ratio between normal vs. impaired awareness during sleeping and waking hours, within the total sample (T1D + T2D, T1D, and T2D), subsectioned by modality of glucose monitoring (CGM + SMBG, CGM, and SMBG users). Benjamini‐Hochberg (BH) correction has been applied for multiple comparisons. *p* < 0.05 have been highlighted as bold.

## Data Availability

The data that support the findings of this study are available from the corresponding author upon reasonable request.
