# Supplementary material for: Factors Associated With Progression From Level 1 to Level 2 Sensor‐Detected Hypoglycaemia in People With Type 1 and Insulin‐Treated Type 2 Diabetes: A Post Hoc Analysis From the Hypo‐METRICS Study
Source: Diabetes Obes Metab. 2026 Aug 4;28(10):9584–94. doi: 10.1111/dom.71172 (PMC13538766; doi:10.1111/dom.71172)
Supplement: Supplementary file 1 [file DOM-28-9584-s001.docx]

**Supplemental table 1**. Characteristics of the study population.

|  | **All diabetes types** | | | **Type 1** | | | **Type 2** | | |
| --- | --- | --- | --- | --- | --- | --- | --- | --- | --- |
| **Variable** | **Type 1**  N = 212*^1^* | **Type 2**  N = 213*^1^* | **p-value***^2^* | **NAH**  N = 167*^1^* | **IAH**  N = 45*^1^* | **p-value***^2^* | **Aware**  N = 162*^1^* | **Unaware**  N = 51*^1^* | **p-value***^3^* |
| Age (yr): median (Q1, Q3) | 49 (30, 58) | 62 (55, 69) | <0.001 | 44 (28, 55) | 53 (49, 63) | <0.001 | 64 (55, 70) | 59 (49, 65) | 0.001 |
| Sex, n (%) |  |  | 0.023 |  |  | 0.5 |  |  | 0.6 |
| Male | 99 (47%) | 123 (58%) |  | 80 (48%) | 19 (42%) |  | 95 (59%) | 28 (55%) |  |
| Female | 113 (53%) | 90 (42%) |  | 87 (52%) | 26 (58%) |  | 67 (41%) | 23 (45%) |  |
| Race, n (%) |  |  | 0.020 |  |  | >0.9 |  |  | 0.3 |
| Other | 8 (3.8%) | 20 (9.4%) |  | 7 (4.2%) | 1 (2.2%) |  | 13 (8.0%) | 7 (14%) |  |
| White | 204 (96%) | 193 (91%) |  | 160 (96%) | 44 (98%) |  | 149 (92%) | 44 (86%) |  |
| Diabetes duration (yr): median (Q1, Q3) | 19 (9, 34) | 18 (13, 25) | 0.5 | 16 (8, 28) | 29 (19, 44) | <0.001 | 20 (14, 26) | 16 (11, 20) | 0.003 |
| Insulin delivery, n (%) |  |  | <0.001 |  |  | 0.7 |  |  | 0.3 |
| MDI | 136 (64%) | 116 (54%) |  | 109 (65%) | 27 (60%) |  | 93 (57%) | 23 (45%) |  |
| Other | 7 (3.3%) | 94 (44%) |  | 6 (3.6%) | 1 (2.2%) |  | 67 (41%) | 27 (53%) |  |
| Pump | 69 (33%) | 3 (1.4%) |  | 52 (31%) | 17 (38%) |  | 2 (1.2%) | 1 (2.0%) |  |
| Glucose monitoring, n (%) |  |  | <0.001 |  |  | 0.051 |  |  | 0.9 |
| CGM | 156 (74%) | 86 (40%) |  | 128 (77%) | 28 (62%) |  | 65 (40%) | 21 (41%) |  |
| SMBG | 56 (26%) | 127 (60%) |  | 39 (23%) | 17 (38%) |  | 97 (60%) | 30 (59%) |  |
| HbA1c (mmol/mol): median (Q1, Q3) | 56 (50, 62) | 58 (51, 67) | 0.014 | 56 (49, 62) | 56 (51, 62) | >0.9 | 58 (51, 67) | 57 (49, 68) | 0.7 |
| Unknown | 3 | 0 |  | 1 | 2 |  |  |  |  |
| IAH, n (%) |  |  | 0.5 | N/A | N/A |  | N/A | N/A |  |
| Aware | 167 (79%) | 162 (76%) |  |  |  |  |  |  |  |
| unaware | 45 (21%) | 51 (24%) |  |  |  |  |  |  |  |
| Mean L1-SDH duration (min): median (Q1, Q3) | 49 (40, 57) | 52 (40, 71) | 0.002 | 48 (39, 56) | 51 (44, 59) | 0.2 | 56 (41, 72) | 49 (38, 63) | 0.15 |
| Mean weekly L1-SDH rate (n/week): median (Q1, Q3) | 5.7 (3.5, 8.0) | 2.4 (1.3, 3.9) | <0.001 | 5.7 (3.5, 8.2) | 5.9 (3.8, 7.8) | 0.8 | 2.42 (1.33, 4.20) | 2.12 (1.31, 3.94) | >0.9 |
| Mean weekly L2-SDH rate (n/week): median (Q1, Q3) | 1.02 (0.40, 2.42) | 0.30 (0.20, 0.65) | <0.001 | 1.01 (0.40, 2.22) | 1.33 (0.61, 3.02) | 0.092 | 0.30 (0.20, 0.71) | 0.30 (0.20, 0.61) | 0.6 |
| Mean weekly Total SDH rate (n/week): median (Q1, Q3) | 7.0 (4.1, 10.6) | 2.7 (1.7, 4.9) | <0.001 | 6.8 (4.0, 10.4) | 7.8 (4.6, 10.8) | 0.3 | 2.73 (1.62, 4.95) | 2.73 (1.82, 4.95) | >0.9 |
| L2 ratio=L2/(total_SDH) (%): median (Q1, Q3) | 16% (10%, 26%) | 14% (9%, 22%) | 0.027 | 16% (10%, 25%) | 19% (13%, 28%) | 0.11 | 13% (9%, 22%) | 16% (10%, 23%) | 0.2 |
| TAR>13.9mmol/L, %: median (Q1, Q3) | 8 (3, 15) | 5 (1, 12) | <0.001 | 8 (4, 16) | 7 (2, 11) | 0.12 | 6 (2, 13) | 3 (1, 10) | 0.13 |
| TAR>10.0mmol/L, %: median (Q1, Q3) | 32 (19, 44) | 28 (14, 42) | 0.054 | 33 (21, 45) | 27 (17, 40) | 0.11 | 30 (16, 42) | 22 (12, 44) | 0.14 |
| TIR, %: median (Q1, Q3) | 61 (52, 72) | 68 (55, 81) | <0.001 | 60 (51, 72) | 62 (55, 74) | 0.2 | 67 (54, 80) | 74 (56, 85) | 0.13 |
| TBR<3.9mmol/L, %: median (Q1, Q3) | 4.5 (2.6, 7.9) | 2.1 (1.1, 4.0) | <0.001 | 4.3 (2.5, 7.8) | 5.5 (2.9, 8.4) | 0.2 | 2.10 (1.10, 4.60) | 2.00 (1.00, 3.80) | 0.7 |
| TBR<3.0mmol/L, %: median (Q1, Q3) | 0.60 (0.20, 1.40) | 0.20 (0.10, 0.50) | <0.001 | 0.50 (0.20, 1.30) | 1.00 (0.30, 2.20) | 0.067 | 0.20 (0.10, 0.50) | 0.20 (0.10, 0.50) | >0.9 |
| Mean glucose (mmol/L): median (Q1, Q3) | 8.60 (7.60, 9.80) | 8.50 (7.50, 9.70) | 0.5 | 8.70 (7.80, 9.90) | 8.30 (7.60, 9.30) | 0.12 | 8.60 (7.70, 9.70) | 8.30 (7.10, 9.80) | 0.2 |
| CV (%): median (Q1, Q3) | 38 (35, 43) | 32 (29, 38) | <0.001 | 38 (34, 43) | 39 (36, 43) | 0.5 | 32.6 (29.4, 37.6) | 31.0 (25.8, 37.6) | 0.11 |
| *^1^*Median (Q1, Q3); n (%) | | | | | | | | | |
| ^2^Wilcoxon rank sum test; Pearson's Chi-squared test | | | | | | | | | |
| *^3^*Wilcoxon rank sum test; Pearson's Chi-squared test; Fisher's exact test | | | | | | | | | |

Note. NAH: normal awareness of hypoglycaemia; IAH: impaired awareness of hypoglycaemia; MDI: multiple daily injections; CGM: continuous glucose monitoring; SMBG: self-monitoring blood glucose; SDH: sensor-detected hypoglycaemia; L1: Level 1 hypoglycaemia (<3.9mmol/L and ≥ 3.0mmol/L); L2: Level 2 hypoglycaemia (<3.0mmol/L); TAR: time above range; TIR: time in range; TBR: time below range; CV: coefficient of variation.

**Supplemental table 2:** Difference in the L2 ratio between sleeping and waking hours within the total sample (T1D+T2D, T1D, and T2D), subsectioned by modality of glucose monitoring (CGM+SMBG, CGM, and SMBG users), and awareness status (NAH+IAH, NAH, IAH). Benjamini-Hochberg (BH) correction has been applied for multiple comparisons. P≥0.05 have been underlined.

| **Type of diabetes** | **Awareness status** | **Mode of glucose monitoring** | **Comparison: Asleep vs Awake** | **L2 ratio: Median (Q1, Q3)** | **p-value*^1^*** | **BH-adjusted**  **p-value** |
| --- | --- | --- | --- | --- | --- | --- |
| T1D+T2D | NAH+IAH | CGM+SMBG | Asleep vs Awake | **21%** (10%, 33%) vs **11%** (3%, 20%) | <0.001 | <0.001 |
| T1D+T2D | NAH | CGM+SMBG | Asleep vs Awake | **21%** (10%, 32%) vs **10%** (3%, 20%) | <0.001 | <0.001 |
| T1D+T2D | IAH | CGM+SMBG | Asleep vs Awake | **24%** (11%, 40%) vs **13%** (4%, 22%) | <0.001 | 0.001 |
| T1D+T2D | NAH+IAH | CGM | Asleep vs Awake | **22%** (10%, 33%) vs **11%** (5%, 22%) | <0.001 | <0.001 |
| T1D+T2D | NAH | CGM | Asleep vs Awake | **22%** (11%, 33%) vs **11%** (4%, 21%) | <0.001 | <0.001 |
| T1D+T2D | IAH | CGM | Asleep vs Awake | **27%** (10%, 40%) vs **14%** (8%, 23%) | 0.01 | 0.01 |
| T1D+T2D | NAH+IAH | SMBG | Asleep vs Awake | **20%** (10%, 31%) vs **9%** (0%, 19%) | <0.001 | <0.001 |
| T1D+T2D | NAH | SMBG | Asleep vs Awake | **18%** (10%, 29%) vs **9%** (0%, 18%) | <0.001 | <0.001 |
| T1D+T2D | IAH | SMBG | Asleep vs Awake | **20%** (11%, 33%) vs **13%** (2%, 21%) | 0.01 | 0.02 |
| T1D | NAH+IAH | CGM+SMBG | Asleep vs Awake | **25%** (13%, 33%) vs **12%** (6%, 23%) | <0.001 | <0.001 |
| T1D | NAH | CGM+SMBG | Asleep vs Awake | **23%** (13%, 33%) vs **11%** (5%, 21%) | <0.001 | <0.001 |
| T1D | IAH | CGM+SMBG | Asleep vs Awake | **28%** (15%, 39%) vs **17%** (10%, 29%) | 0.03 | 0.04 |
| T1D | NAH+IAH | CGM | Asleep vs Awake | **25%** (13%, 33%) vs **12%** (6% 23%) | <0.001 | <0.001 |
| T1D | NAH | CGM | Asleep vs Awake | **24%** (12%, 33%) vs **12%** (5%, 23%) | <0.001 | <0.001 |
| T1D | IAH | CGM | Asleep vs Awake | **31%** (20%, 43%) vs **15%** (10%, 28%) | 0.007 | 0.009 |
| T1D | NAH+IAH | SMBG | Asleep vs Awake | **23%** (12%, 33%) vs **11%** (6%, 21%) | 0.002 | 0.003 |
| T1D | NAH | SMBG | Asleep vs Awake | **23%** (13%, 33%) vs **11%** (5%, 17%) | <0.001 | <0.001 |
| T1D | IAH | SMBG | Asleep vs Awake | **20%** (8%, 32%) vs **21%** (7%, 29%) | >0.9 | >0.9 |
| T2D | NAH+IAH | CGM+SMBG | Asleep vs Awake | **17%** (9%, 29%) vs **8%** (0%, 18%) | <0.001 | <0.001 |
| T2D | NAH | CGM+SMBG | Asleep vs Awake | **17%** (9%, 27%) vs **8%** (0%, 17%) | <0.001 | <0.001 |
| T2D | IAH | CGM+SMBG | Asleep vs Awake | **20%** (7%, 38%) vs **11%** (1%, 19%) | 0.003 | 0.004 |
| T2D | NAH+IAH | CGM | Asleep vs Awake | **16%** (8%, 26%)  vs **9%** (3%, 17%) | 0.001 | 0.001 |
| T2D | NAH | CGM | Asleep vs Awake | **17%** (9%, 29%) vs **9%** (0%, 17%) | 0.001 | 0.001 |
| T2D | IAH | CGM | Asleep vs Awake | **18%** (3%, 40%) vs **13%** (4%, 20%) | 0.43 | 0.45 |
| T2D | NAH+IAH | SMBG | Asleep vs Awake | **17%** (9%, 29%) vs **8%** (0%, 18%) | <0.001 | <0.001 |
| T2D | NAH | SMBG | Asleep vs Awake | **17%** (8%, 29%) vs **7%** (0%, 20%) | <0.001 | <0.001 |
| T2D | IAH | SMBG | Asleep vs Awake | **21%** (16%, 33%) vs **10%** (0%, 16%) | 0.001 | 0.002 |
| *^1^*Wilcoxon rank-sum test |  |  |  |  |  |  |

Note. NAH: normal awareness of hypoglycaemia; IAH: impaired awareness of hypoglycaemia; CGM: continuous glucose monitoring; SMBG: self-monitoring blood glucose; L2 ratio: (L2-SDH)/(total-SDH), with total-SDH defined as the aggregate of L1 and L2-SDH events; SDH: sensor-detected hypoglycaemia.

**Supplemental table 3:** Difference in the L2 ratio between normal vs impaired awareness during sleeping and waking hours, within the total sample (T1D+T2D, T1D, and T2D), subsectioned by modality of glucose monitoring (CGM+SMBG, CGM, and SMBG users). Benjamini-Hochberg (BH) correction has been applied for multiple comparisons. *P*<0.05 have been highlighted as bold.

| **Type of diabetes** | **Sleep/awake status** | **Mode of glucose monitoring** | **Comparison: NAH vs IAH** | **L2 ratio: Median (Q1, Q3)** | **p-value*^1^*** | **BH-adjusted**  **p-value** |
| --- | --- | --- | --- | --- | --- | --- |
| T1D+T2D | Asleep+awake | CGM+SMBG | NAH vs IAH | **14%** (6%, 26%) vs **17%** (7%, 31%) | **0.038** | 0.2 |
| T1D+T2D | Asleep | CGM+SMBG | NAH vs IAH | **21%** (10%, 32%) vs **24%** (11%, 40%) | 0.17 | 0.36 |
| T1D+T2D | Awake | CGM+SMBG | NAH vs IAH | **10%** (3%, 20%) vs **13%** (4%, 22%) | **0.045** | 0.2 |
| T1D+T2D | Asleep+awake | CGM | NAH vs IAH | **15%** (7%, 27%) vs **20%** (9%, 34%) | 0.056 | 0.2 |
| T1D+T2D | Asleep | CGM | NAH vs IAH | **22%** (11%, 33%) vs **27%** (10%, 40%) | 0.17 | 0.36 |
| T1D+T2D | Awake | CGM | NAH vs IAH | **11%** (4%, 21%) vs **14%** (8%, 23%) | 0.06 | 0.2 |
| T1D+T2D | Asleep+awake | SMBG | NAH vs IAH | **13%** (5%, 25%) vs **17%** (5%, 26%) | 0.24 | 0.45 |
| T1D+T2D | Asleep | SMBG | NAH vs IAH | **18%** (10%, 29%) vs **20%** (11%, 33%) | 0.46 | 0.53 |
| T1D+T2D | Awake | SMBG | NAH vs IAH | **9%** (0%, 18%) vs **13%** (2%, 21%) | 0.31 | 0.48 |
| T1D | Asleep+awake | CGM+SMBG | NAH vs IAH | **17%** (8%, 28%) vs **22%** (10%, 34%) | **0.02** | 0.18 |
| T1D | Asleep | CGM+SMBG | NAH vs IAH | **23%** (13%, 33%) vs **28%** (15%, 39%) | 0.25 | 0.45 |
| T1D | Awake | CGM+SMBG | NAH vs IAH | **11%** (5%, 21%) vs **17%** (10%, 29%) | **0.02** | 0.18 |
| T1D | Asleep+awake | CGM | NAH vs IAH | **17%** (8%, 30%) vs **23%** (12%, 35%) | **0.01** | 0.18 |
| T1D | Asleep | CGM | NAH vs IAH | **24%** (12%, 33%) vs **31%** (20%, 43%) | **0.04** | 0.2 |
| T1D | Awake | CGM | NAH vs IAH | **12%** (5%, 23%) vs **15%** (10%, 28%) | 0.07 | 0.22 |
| T1D | Asleep+awake | SMBG | NAH vs IAH | **15%** (8%, 25%) vs **21%** (7%, 31%) | 0.62 | 0.7 |
| T1D | Asleep | SMBG | NAH vs IAH | **23%** (13%, 33%) vs **20%** (8%, 32%) | 0.37 | 0.48 |
| T1D | Awake | SMBG | NAH vs IAH | **11%** (5%, 17%) vs **21%** (7%, 29%) | 0.09 | 0.23 |
| T2D | Asleep+awake | CGM+SMBG | NAH vs IAH | **12%** (4%, 25%) vs **16%** (4%, 25%) | 0.38 | 0.48 |
| T2D | Asleep | CGM+SMBG | NAH vs IAH | **17%** (9%, 27%) vs **20%** (7%, 38%) | 0.36 | 0.48 |
| T2D | Awake | CGM+SMBG | NAH vs IAH | **8%** (0%,17%) vs **11%** (1%, 19%) | 0.45 | 0.53 |
| T2D | Asleep+awake | CGM | NAH vs IAH | **12%** (6%, 25%) vs **16%** (4%, 26%) | 0.85 | 0.88 |
| T2D | Asleep | CGM | NAH vs IAH | **17%** (10%, 25%) vs **18%** (3%, 40%) | 0.75 | 0.81 |
| T2D | Awake | CGM | NAH vs IAH | **8%** (0%, 15%) vs **13%** (4%,20%) | 0.28 | 0.47 |
| T2D | Asleep+awake | SMBG | NAH vs IAH | **12%** (2%, 24%) vs **16%** (4%, 25%) | 0.35 | 0.48 |
| T2D | Asleep | SMBG | NAH vs IAH | **17%** (8%, 29%) vs **21%** (16%, 33%) | 0.14 | 0.34 |
| T2D | Awake | SMBG | NAH vs IAH | **7%** (0%, 20%) vs **10%** (0%, 16%) | 0.95 | 0.95 |
| *^1^*Wilcoxon rank-sum test |  |  |  |  |  |  |

Note. NAH: normal awareness of hypoglycaemia; IAH: impaired awareness of hypoglycaemia; CGM: continuous glucose monitoring; SMBG: self-monitoring blood glucose; L2 ratio: (L2-SDH)/(total-SDH), with total-SDH defined as the aggregate of L1 and L2-SDH events; SDH: sensor-detected hypoglycaemia.
